# Treatment of Erysipelas in the Limbs by Elevation

**Published:** 1859-09

**Authors:** M. Henry


*Treatment of Erysipelas in the Limbs by Elevation, by M. Henry, Esq.*

We have noticed a very useful plan of treatment for erysipelas of the extremities, adopted by Mr. Mitchell Henry, at the Middlesex Hospital, which is worthy of a fair trial elsewhere. It consists in elevating the affected leg or arm in a vertical direction, above the horizontal plane of the body. This causes a subsidence of the swelling associated with the disease, and completely removes the pain; the circulation in the veins is accelerated towards the heart, and the hitherto inflamed and red skin assumes a pallid aspect. All these good results we witnessed, on the 3d of December, in a very severe case of erysipelas attacking the left leg of an elderly man, who suffered most severely from acute pain consequent on the swelling of the limb from the inflammation. In twelve hours both the pain and the swelling had entirely disappeared under this very simple mode of treatment. The same good effects had also ensued in the case of erysipelas of the elbow in a boy who was pointed out to us. The limbs, especially the inferior, may be supported on pillows, but it is more suitable to elevate them by the hand or the foot by means of a cord attached to the framework of the patient's bed.—*Lancet, Dec.* 18, *p.* 631.

